# Sleep–Wake Dysregulation in Human African Trypanosomiasis: From Neuroinvasion to Neuronal Dysfunction

**DOI:** 10.3390/clockssleep8030042

**Published:** 2026-07-08

**Authors:** Seithikurippu R. Pandi-Perumal, Ahmed S. BaHammam, Konda Mani Saravanan

**Affiliations:** 1 Centre for Research and Development, Chandigarh University, Mohali 140413, Punjab, India; pandiperumal2023@gmail.com; 2 Division of Research and Development, Lovely Professional University, Phagwara 144411, Punjab, India; 3 University Sleep Disorders Center, Department of Medicine, College of Medicine, King Saud University, Riyadh 11324, Saudi Arabia; ashammam2@gmail.com; 4 Centre for Research Impact & Outcome, Chitkara College of Pharmacy, Chitkara University, Rajpura 140401, Punjab, India; 5 Center for Innovation and Inclusive Research, Sharda University, Greater Noida 201310, Uttar Pradesh, India

**Keywords:** human african trypanosomiasis, sleeping sickness, sleep–wake disorders, circadian rhythms, neuroinflammation, neuroinvasion, suprachiasmatic nucleus, trypanosome-associated sleep disorder

## Abstract

Human African trypanosomiasis (HAT) or sleeping sickness is a neglected tropical disease with a progressive central nervous system (CNS) involvement and marked sleep and circadian rhythm abnormalities. Even though this is a prominent feature of HAT, the connection between parasite neuroinvasion, neuroinflammation, circadian dysfunction, and neurological impairment is not fully understood. This narrative review aims to summarize the most up-to-date knowledge about sleep and circadian disturbance in HAT and proposes an integrated approach for the Trypanosome-Associated Sleep Disorder (TASD). The relevant literature was identified by searching major biomedical databases for HAT, sleep disorders, circadian rhythms, neuroinflammation, and CNS invasion. The review covers the steps by which the CNS becomes invaded, how the barriers are disrupted, how the CNS becomes activated by inflammatory responses, and how the hypothalamic and circadian regulatory networks are disrupted. The evidence suggests that excessive daytime sleepiness, fragmented nocturnal sleep, circadian misalignment, and neuropsychiatric manifestations are related to the activation of inflammatory cytokines, altered neurotransmitter signaling, activation of the kynurenine pathway, dysregulation of clock genes, and disruption of the suprachiasmatic nucleus. We also discuss TASD as a syndrome-like phenotype of CNS involvement and propose a three-stage model of sleep–wake dysfunction in HAT. The review unites these integral mechanisms in a single mechanistic framework to offer a unified understanding of the sleep pathology associated with HAT. There are still important gaps in our knowledge of biomarkers, disease staging, and irreversible neuronal damage, which indicate priorities for future research and better clinical management.

## 1. Introduction

Human African trypanosomiasis (HAT), also referred to as sleeping sickness, shows how a parasitic infection can progressively reshape the neural control of sleep and circadian timing [1]. HAT is a serious disease caused by a protozoan parasite [2]. Recent advances in understanding HAT pathophysiology have revealed that sleep–wake dysregulation represents not merely a symptom but a core pathophysiological feature driven by specific disruptions in hypothalamic circuitry and circadian networks. Transmitted by the tsetse fly, *Trypanosoma brucei* has ravaged sub-Saharan Africa, serving not only as a public health crisis but also as a fundamental paradigm that has informed our comprehension of neuroinfectious diseases [3]. On top of its epidemiological significance, sleeping sickness is in a special position in biomedical science since the pathogenic symptom is the disruption of sleep and circadian organization [4,5]. Unlike many neurotropic pathogens that produce coma, seizures, or focal neurological deficits as primary or early manifestations, *T. brucei* infection uniquely drives a progressive disruption of sleep–wake architecture, while seizures and similar central nervous system (CNS) signs are late-stage features [6,7,8]. This distinctive phenotype has attracted clinical and neuroscientific attention. However, the underlying mechanisms have only recently begun to be elucidated. This single phenotype has long attracted the attention of clinicians and neuroscientists, and the cellular and circuit-level mechanisms underlying this phenotype are now being defined [9,10].

**Figure 1:**
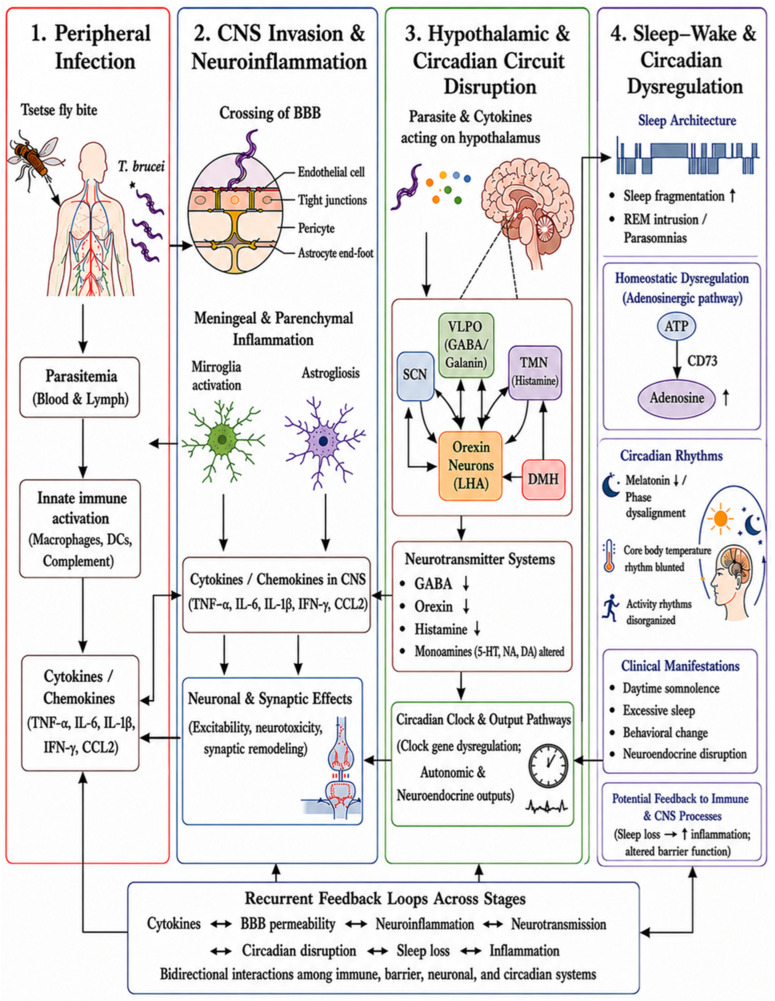
Proposed mechanistic framework linking *Trypanosoma brucei* infection to sleep–wake and circadian dysregulation in HAT. Following tsetse fly transmission, *Trypanosoma brucei* infection triggers systemic immune activation and neuroinflammation, facilitating CNS invasion. Inflammatory and parasite-derived factors disrupt blood–brain barrier integrity and alter hypothalamic and circadian regulatory circuits. These changes contribute to sleep fragmentation, REM sleep abnormalities, excessive daytime sleepiness, and circadian rhythm disruption. Bidirectional interactions among immune, barrier, neural, and circadian mechanisms are shown to highlight the complex, non-linear pathogenesis of HAT-associated sleep dysfunction. Figure prepared by the authors with the assistance of generative AI-based drawing tools. All scientific content was conceived, reviewed, and verified by the authors, who take full responsibility for its accuracy.

The initial definitions of sleeping sickness focused on somnolence, lack of energy, and change in behavior and tended to simplify the disease as an excessive daytime sleepiness disorder [11]. The following clinical, electrophysiological, and neuroendocrine investigations have shown a much more complicated picture. Patients experience not only increased sleep duration but also a loss of the temporal arrangement between sleep and wakefulness [9]. For example, Rapid Eye Movement (REM) sleep may occur during sleep onset (SOREMPs, Sleep-Onset REM Periods), and circadian rhythms that govern hormones, body temperature, and activity are significantly disorganized [12]. These changes represent a profound perturbation of the central timekeeping and sleep–wake systems and involve hypothalamic, brainstem, and limbic pathways, which combine metabolic cues with immune and environmental stress [13]. In addition to circadian dysregulation, growing evidence suggests that HAT also disrupts the homeostatic regulation of sleep (Process S) [14]. Rijo-Ferreira and colleagues demonstrated that trypanosome infection alters adenosine signaling, potentially through increased extracellular adenosine accumulation mediated by ecto-5′-nucleotidase (CD73)-dependent pathways [15]. Given the central role of adenosine in sleep pressure accumulation, these findings indicate that excessive sleepiness in HAT may arise not only from circadian clock disruption (Process C) but also from impaired sleep homeostasis. Viewed through the two-process model of sleep regulation, Trypanosome-Associated Sleep Disorder (TASD) may therefore represent a unique condition in which both circadian and homeostatic mechanisms are simultaneously compromised, contributing to the characteristic combination of daytime somnolence, fragmented nocturnal sleep, and altered REM sleep regulation. TASD is a disruption of the 24 h sleep–wake rhythm that gives the disease its common name. Readers should note that the International Classification of Sleep Disorders, third edition, Text Revision (ICSD-3-TR) references TASD in the following context: “In the context of trauma and Post-Traumatic Stress Disorder (PTSD), REM sleep Behavior Disorder (RBD) and REM sleep Without Atonia (RWA) have been reported, and the term trauma-associated sleep disorder (TASD/TSD) has been proposed (page 303)” (American Academy of Sleep Medicine, 2023) [16], which diverges significantly from the current discussion. Therefore, HAT serves as a unique natural model for studying the biological basis of sleep and circadian control during pathological states [10]. The proposed pathways linking peripheral infection to neurological and sleep disturbances are presented in Figure 1.

The neurologic stage of HAT is characterized by an intrusion of the parasite into the CNS that changes a systemic hemolymphatic infection into a diffuse meningoencephalitis [17]. This change is of clinical importance, as it determines treatment options, prognosis, and mortality [18]. Neuroinvasion, in a mechanistic approach, poses inherent questions of how a huge extracellular organism invades protective barriers like the blood–brain barrier (BBB) and blood–cerebrospinal fluid barrier (BCSFB) and survives in the hostile brain environment, and how neural functions are restructured without directly infecting the neurons [19]. It is increasingly becoming clear that *T. brucei* takes advantage of both anatomical and host inflammatory responses and employs a complex of parasite-derived enzymes, host matrix remodeling, and immune cell traffic to access the CNS [20]. The parasite induces a long-lasting neuroinflammatory reaction that turns out to become one of the key generators of neuropathology [21]. Sleeping sickness is marked by the widespread activation of microglia and astrocytes, inflammation by peripheral immune cells, and a complex cytokine and chemokine environment, dominated by interferon-γ, tumor necrosis factor-α (TNF-α), and chemokines like CXCL10 [22]. Although this type of response is important to control parasites, it has significant neuromodulatory effects [23]. The cytokines and the inflammatory genes are now discussed as powerful regulators of sleep and circadian cycles, able to adjust the neuronal excitability, synaptic plasticity, and neurotransmitter equilibrium [24]. In this regard, the prominent sleeping disorders of HAT could be considered not a collateral event but a collective feature of immune/neural crosstalk in the brain of the infected host [15]. This view identifies sleeping sickness as a disease where inflammation alters behavior and cognition, such as autoimmune encephalitis, neurodegenerative disease, and chronic infection [25].

On the molecular front, numerous pathways collaborate to disrupt sleep–wake regulation in the process of *T. brucei* infection. Available experimental data indicate that metabolites produced by parasites, especially those produced on the basis of the host tryptophan metabolism, may contribute to dysregulation of neuronal networks [26]. At the same time, the inflammatory mediators like interleukin-1 and TNF-α are endogenous somnogens, which change the volume and the quality of sleep [27]. Aminergic neurotransmitter systems, such as serotonin and dopamine, also disrupt the sleep–wake balance [28]. An additional level of neuromodulation is the overproduction of nitric oxide and oxidative stress that connects the activation of immunity to alteration of synaptic signaling and neuronal health [29]. These observations show that sleep disruption in sleeping sickness emerges from the interaction between parasite persistence, host immune activation, and altered neuronal signaling. Although the pathogenesis of this disease has been studied over time, significant diagnostic and treatment problems remain [30]. The CNS involvement is still very challenging to detect based on flawed cerebrospinal fluid (CSF) indicators and invasive examinations [31]. Conventional modalities of late-stage disease treatment need to be able to penetrate the brain well enough, but are frequently constrained by toxicity and risk of treatment-related encephalopathy [32]. The results of a successful parasite clearance of the host organism in many cases include lasting neurological and cognitive impairment, which supports the rationale of implementing neuroprotective approaches beyond antiparasitic efficacy [33]. All these questions remain unanswered, which underlines the fact that sleeping sickness is not only an infectious disease but also a chronic disturbance of the CNS [34]. The persistence of neurological deficits in a subset of treated patients underscores the importance of early diagnosis and raises questions about whether adjunctive neuroprotective strategies could improve long-term outcomes.

This narrative review collects the knowledge available on sleep–wake disturbances in HAT. The literature search was carried out in the Web of Science and SCOPUS databases from 15 to 31 January 2026. The search strategy was a combination of the following keywords and variants with the Boolean operators: African trypanosomiasis, sleeping sickness, *Trypanosoma brucei*, sleep, circadian rhythm, sleep–wake cycle, neuroinflammation, suprachiasmatic nucleus (SCN), orexin, and hypothalamus. Only articles that were published in English were considered. Relevant peer-reviewed original research articles, systematic and narrative reviews, and authoritative clinical and public health reports were identified. Additional papers were found using backward and forward citation tracking from key papers. However, studies were selected based on their relevance to the aims of this review, and preference was given to studies that contained mechanistic information about changes in sleep and in circadian regulation induced by the parasites of HAT. Clinical studies that reported sleep-related changes in HAT, experimental animal studies that added to the understanding of the pathophysiological pathways, and historical studies that led to the establishment of foundational concepts in the field were considered. Most studies not directly pertinent to sleep–wake regulation, the circadian biology, and neurobiological mechanisms of HAT were not discussed in detail.

In this connection, the present review seeks to summarize the information about the neurobiological mechanism of sleep dysregulation in HAT by following the disease pathology from the peripheral infection to the CNS invasion and functional impairment. Our literature review focuses on the clinical presentation of TASD and the circadian rhythm associated with it. The manner in which *T. brucei* invades CNS barriers to induce neuroinflammation is thoroughly described. We then discuss the cellular and molecular pathways connecting the activation of the immune and parasite-derived factors in the regulation of sleep, followed by a discussion of the diagnostic limitations and treatment implications. Finally, we outline the important unresolved questions and perspectives and underscore the utility of sleeping sickness as a paradigm to learn how infection and inflammation reorganize the basic brain conditions like sleep.

## 2. Sleep–Wake Dysregulation in HAT

HAT is a clinical disorder characterized by an extreme and progressive impairment of sleep–wake regulation. However, it is a complex disturbance of the circadian timing, the structure of sleep, and the stability of vigilance that is the hallmark of the disease, rather than just plain hypersomnolence [35]. These changes are indicative of direct invasion of the CNS by trypanosomes, neuroinflammation, and pathophysiology of hypothalamic and brainstem circuits that control sleep, wake, and the biological timing system [36]. Together, these changes define the sleep and circadian phenotype described here as TASD [37]. Although TASD is not yet formally recognized in major diagnostic classifications, it provides a useful conceptual framework for understanding the distinct sleep–wake phenotype in HAT.

In this review, TASD is not a formal diagnostic entity, but rather a descriptive construct. TASD includes daytime sleepiness, nocturnal sleep fragmentation, disruption of circadian rhythmicity, changes in sleep architecture, and the presence of SOREMPs, which are all characteristic abnormalities of sleep and circadian rhythms seen in HAT. These characteristics differentiate sleep disturbance in HAT from classical sleep disorders (e.g., narcolepsy and idiopathic hypersomnia), but the evidence base for TASD as a recognized diagnostic category is limited. Furthermore, different clinical manifestations are reported from a mix of patients, disease stage, and methods of evaluation. For now, TASD should be considered a syndrome-like phenotype of CNS involvement in HAT rather than a nosological entity. Future research using standardized clinical evaluations and polysomnographic recordings, as well as circadian and disease-staging parameters, will be required to establish consensus diagnostic criteria and to assess the need for the inclusion of TASD in the formal classification of sleep disorders as a unique entity.

The suggested comparison shown in Table 1 emphasizes that TASD has excessive daytime sleepiness and sleep-onset REM periods as seen in narcolepsy type 1, but is distinct in that it is a secondary, infection-driven disease with a prominent neuro-inflammatory profile. TASD is characterized by CSF pleocytosis, increased levels of intrathecal inflammatory markers (Immunoglobulin M (IgM), neopterin, chemokine (CXCL13)), and significant disruption of cortisol, melatonin, and core body temperature circadian rhythms, which are not seen in narcolepsy type 1 and idiopathic hypersomnia. All of these clinical, physiological, and biological features are distinctive and reinforce the view that the syndrome of TASD is a separate form of secondary hypersomnolence related to HAT and that the proposed criteria are provisional and require validation in prospective clinical studies.

**Figure 2:**
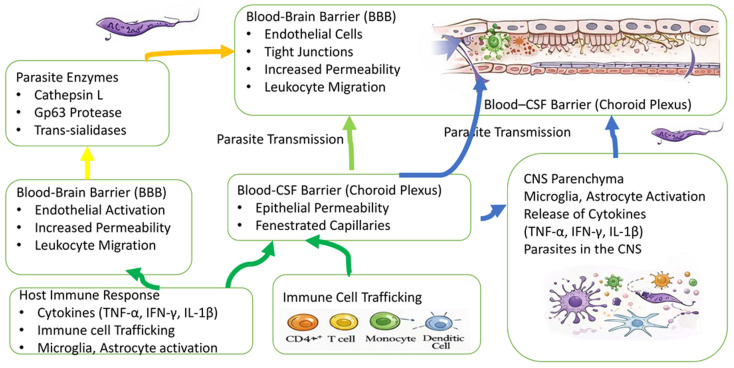
Mechanisms of CNS invasion and immune activation in HAT. Figure prepared by the authors with the assistance of generative AI-based drawing tools. All scientific content was conceived, reviewed, and verified by the authors, who take full responsibility for its accuracy.

Patients with TASD develop the inability to regulate the temporal integration of sleep and wakefulness into distinct periods. Naps during the day are inappropriate and too frequent, whereas nighttime sleep turns discontinuous and not restful [38]. Patients can sleep in the middle of discussion, eating, or performing physical exercise, and be awake or excited during the night. This trend is an expression of instability of the vigilance control and not a higher physiological necessity of sleep [39]. These abnormalities develop dynamically with the progression of the disease and worsen with the development of parasites penetrating the BBB and being concentrated in the CNS, which is observed through longitudinal clinical observations [40]. The breakdown of CNS barriers during infection, driven by parasite enzymes and immune cell trafficking, is depicted in Figure 2.

While TASD shares several clinical and polysomnographic characteristics with narcolepsy, such as excessive daytime sleepiness (EDS), SOREMPs, and orexinergic signaling dysfunction, there is currently evidence that there are important mechanistic differences. Experimental research in HAT has shown that there is damage to orexin-producing neurons and disruption of hypothalamic sleep–wake regulatory networks, but the amount of orexinergic neuronal loss and the differential involvement of orexin receptor subtypes are still poorly characterized [36]. In addition, sleep–wake abnormalities in HAT occur in the background of increased neuroinflammation, circadian abnormalities, and parasite-induced neuropathology, as opposed to the selective autoimmune destruction of orexin neurons seen in narcolepsy. Inflammatory and parasitic processes may directly or indirectly affect key neural circuits involved in vigilance regulation, such as the lateral hypothalamic area, the ventrolateral preoptic nucleus, and the locus coeruleus, which may account for the complex nature of sleep in HAT. It is suggested that TASD may serve as a useful concept to guide the integration of the unique sleep and circadian disturbances found in trypanosome infection. Further prospective clinical and mechanistic studies are needed to validate the neurobiological basis and diagnostic classification of TASD.

Recording objective sleep shows that TASD is essentially different from such primary sleep disorders as narcolepsy or idiopathic hypersomnia. Despite the presence of SOREMPs, TASD is devoid of the stable phenotype, genetic predisposition, and autoimmune characteristics of narcolepsy [41]. Rather, sleep–wake interference is associated with CSF pleocytosis, the increase in inflammatory mediators, and neurological stages of the disease [42]. These results validate the definition of TASD as an infection-induced secondary disorder of sleep–wake regulation. One of the critical symptoms of TASD is gross circadian rhythm alteration [43]. In a healthy environment, circadian pacemakers based in the SCN synchronize sleep schedule with endocrine release, body temperature, and behavioral activity. This coordination gradually worsens in HAT. The circadian cortisol rhythms that are usually high in the early morning hours to keep one alert are flattened, phase-shifted, or even arrhythmic [12]. This impaired rhythmic cortisol secretion helps in causing daytime sleepiness, poor responses to stress, and metabolic dysregulation [44]. Equally, melatonin secretion levels, which normally increase at night to facilitate sleep onset and provide circadian timing cues, demonstrate significant alterations during HAT infection. Experimental studies in rat models of African trypanosomiasis have revealed disruptions in melatonin secretion patterns and alterations in melatonin receptor binding within the SCN, indicating fundamental disruption of the pineal–SCN axis [44,45]. In human patients, melatonin secretion patterns tend to be out of phase with the light–dark (LD) cycle.

Other strong indicators of circadian integrity, such as core body temperature (CBT) rhythms, are also disturbed [24]. A delayed and clustered amplitude or oscillations throughout the night and day are observed in place of a steady nocturnal fall and a daytime rise. The fact that hormonal, thermal, and behavioral rhythms are uncoupled suggests central impairment of circadian control and not just peripheral unsynchrony [46]. Through experimental and clinical evidence, it has been proposed that the inflammatory activity in hypothalamic areas such as the SCN and other neighboring nuclei disrupts neuronal firing activities and expression of clock genes [47]. Increased production of pro-inflammatory cytokines in the CSF also destabilizes circadian products, which makes active immune reactions directly related to disrupting the rhythm [48].

Parallel to the circadian abnormalities, TASD is also characterized by far-reaching changes in sleep architecture [49]. Polysomnographic investigations mostly reveal severe fragmentation in non-REM and REM sleep, and changes between vigilance states occur regularly [50]. Sleep is poorly consolidated and shallow, which causes low efficiency of sleep, even though there is a long duration of time spent in bed. Among the most apparent abnormalities is the common occurrence of SOREMPs when the REM sleep intrudes immediately or soon after the onset of sleep. This is an indication of dysfunction in brainstem monoaminergic systems that normally inhibit REM sleep in the state of wakefulness, probably because of trypanosome-induced inflammation and neuronal dysfunction in areas of the locus coeruleus (LC), dorsal raphe nucleus (DRN), and pedunculopontine tegmentum (PPT) [51].

Later in the progression of the disease, a significant proportion of patients exhibit a severe disturbance in the sleep–wake cycle, where the patients fall asleep during the day and stay awake at night [52]. This is in contrast to adaptive circadian changes observed in the case of shift work or jet lag, which is a fluctuating and inefficient inversion with bouts of sleep distributed non-uniformly throughout the 24-h day. These changes may be insufficiently known to patients, which also makes it harder to clinically assess them. This leads to disturbance of cognitive functions, motor coordination, and alertness that makes accidents and functional impairment very likely to occur, especially in resource-constrained endemic conditions [53]. Sleep–wake disorders in HAT have a close relationship with a wide range of neurological and neuropsychiatric manifestations. At the onset of the disease, the patient might have the nonspecific symptoms of headaches, fatigue, irritability, and decreased concentration [48]. As the condition advances with the involvement of the CNS, some more serious characteristics manifest themselves in the form of tremors, gait instability, seizures, and changes in the state of consciousness. There are neuropsychiatric symptoms of particular concern, such as depression, anxiety, personality disorders, hallucinations, and psychosis [54]. These phenotypes are frequently compounded by persistent sleep fragmentation and circadian misalignment, which form a reciprocating association between disruption of sleep and neurobehavioral malfunction [55].

These clinical characteristics are indicators of generalized neuroinflammation, gliosis, and disturbance of neurotransmitter systems that control sleep and wakefulness at the mechanistic level [56]. The changes in dopaminergic, serotonergic, and cholinergic signaling have a role in causing cognitive impairment, emotional instabilities, and perceptual disturbances. Notably, most sleep and neuropsychiatric abnormalities are partially reversible with effective antiparasitic treatment, especially when treatment is started before a significant amount of neuronal damage has been inflicted [57]. These neurochemical and behavioral neurobiological defects occur only upon entering the CNS of the parasite. The shift of peripheral infection to the brain is one of the critical points in the disease progression, and the process of pathological events that support the evolution of sleep–wake disturbance and neuropsychiatric impairment starts [32]. The mechanisms by which *T. brucei* passes through protective brain barriers are therefore key to the understanding of the neurological stage of HAT.

## 3. Mechanisms of Neuroinvasion

The hypothesis-generating approach of the three-stage TASD model proposed here should be understood as such, considering the existing evidence on the temporal evolution of the sleep and circadian disturbances in the context of HAT. Stage 1 involves peripheral infection and immune system activation, which will likely disrupt sleep architecture via cytokines and inflammatory mediators in the bloodstream, causing subtle disturbances to the circadian timing and sleep–wake regulation. In stage 2, the progressive neuroinflammation, BBB dysfunction, and hypothalamic involvement are associated with the appearance of TASD symptoms, including excessive daytime sleepiness, fragmented nocturnal sleep, and sleep onset REM periods. Stage 3 is in the advanced stages of neuropathology and may involve ongoing inflammation, neuronal dysfunction, and damage to sleep-regulatory circuits that can result in ongoing and permanent sleep deficits. While experimental studies have suggested a role for the inflammatory mediators IFN-γ, TNF-α, IL-1β, and the matrix metalloproteinases in HAT neuropathogenesis, direct evidence for the connection between modulation of these pathways and prevention of progression of TASD is limited. Thus, our hypothesis that early anti-inflammatory or neuroprotective treatment might be able to slow the progression from Stage 1 or Stage 2 to Stage 3 should be considered a prediction to be tested in clinical trials and not a therapeutic guideline [58]. Longitudinal animal models and prospective clinical cohorts should be used to determine if specific inflammatory pathways could be targeted to prevent severe TASD manifestations, or if protection of the BBB integrity or sleep-regulatory networks in the hypothalamus and/or brainstem can prevent severe TASD manifestations. These investigations would offer a vital context for the validity of the proposed staging model and its translational implications, as well as for its refinement and challenge.

Transformation of peripheral infection into the CNS is one of the events that characterize the development of HAT. This transformation may be regarded as the beginning of the meningoencephalitic stage and is the basis of the development of sleep–wake disturbance, neuropsychiatric signs, and permanent neurological damage [33]. Key to this shift is that the parasite has managed to overcome extremely specialized brain barriers, which typically help the neural milieu to resist the entry of circulating pathogens and immune responder cells [59]. Instead of being a one-time disastrous event, neuroinvasion is a multiphase process in which the initial phase is early entry into the CSF chambers, which is followed by continued destabilization of the parenchymal BBB [60]. The brain is safeguarded by two main anatomical interfaces, the BBB and the BCSFB. Negative endothelial cells attached by tight junctions form the BBB, surrounded by pericytes and astrocytic end-feet, and optimally regulate the movement of molecules and cells into the brain parenchyma [61]. Conversely, the BCSFB is concentrated mainly in the choroid plexus, which has fenestrated capillaries enveloped by a polarized epithelial cell layer, which are held together by tight junctions [62]. Although both barriers have a gatekeeping role, the choroid plexus is a relatively open and immunologically active structure, making it a probable point of entry at initial phases of neuroinvasion [63]. It is important to note that while experimental models using *T. brucei* in rodents have provided invaluable mechanistic insights, there are inherent limitations in extrapolating these findings to human *T. b. gambiense* infection. As demonstrated by Tesoriero et al. (2018) [47], *T. b. gambiense* exhibits greater neurotropism and causes more substantial SCN neuronal loss compared to *T. brucei*, suggesting that human disease pathology may be underestimated by conventional animal models [64]. Moreover, the chronic, multi-year course of *T. b. gambiense* HAT in humans differs markedly from the weeks-to-months-long progression in rodent *T. brucei* infections, potentially involving additional or distinct pathophysiological mechanisms. The way in which *T. brucei* can enter the CNS is not fully understood, and experimental data indicate that several, possibly synergistic, mechanisms are involved in neuroinvasion [65]. Experimental studies of parasites and cerebral endothelial cells, and the ability of trypanosomes to cross barriers using parasite-derived proteases and host inflammatory mediators, suggest that one mechanism of direct transmigration may occur across the BBB [66]. This model is further reinforced by the observation that parasites can be seen in the brain-associated vascular compartments before the onset of extensive leukocyte infiltration. In contrast, the “Trojan Horse” hypothesis is that activated leukocytes can function as vehicles to enter the CNS, especially when the BBB is permeable during inflammation [67]. Supporting evidence for this mechanism is the temporal relationship between the recruitment of immune cells, activation of endothelium by cytokines, and the accumulation of parasites in the CNS [68]. Despite this, in vivo visualization of leukocyte-mediated parasite transport is still limited. All these mechanisms can occur sequentially in various phases of infection or in different inflammatory contexts. Thus, current evidence suggests the multifactorial model of neuroinvasion in which direct migration of the parasite, immune-mediated barrier remodeling, and leukocyte-associated transport all contribute to invasion of the CNS and disease progression. The stepwise mechanisms and molecular actors facilitating CNS invasion are detailed in Table 2.

One major factor that complicates the literature on sleep–wake disturbances in HAT is that there is significant variability in experimental systems and parasite species used to collect the data that are available. Most human disease is caused by *T. b. gambiense* and, less frequently, by *T. b. rhodesiense*, while most of the understanding of the mechanisms of neuroinvasion, neuroinflammation, circadian disruption, and sleep pathology has come from experimental infections of rodents [36]. These animal models have been extremely useful for understanding the interactions between parasites and host as well as the pathology of the central nervous system, but have not been able to fully recapitulate the chronic course of disease, neuropathological changes, and sleep–wake abnormalities seen in humans infected with these organisms. In addition, parasite virulence, host immune responses, and sensitivity of sleep-regulatory structures such as the SCN may affect experimental results, given the species-specific differences. Thus, the results obtained from animal models should be used with great caution and should be viewed as mostly mechanistic and not as a representation of the human disease.

The importance of disruption of brain microvascular endothelial integrity by parasite-derived cysteine proteases, particularly bruceipain, is increasingly apparent in ultrastructural and in vitro studies [74]. Experimental exposure of brain endothelial cells to *T. brucei* or purified bruceipain activates the endothelial cells and leads to increased endothelial permeability and changes in distinct tight junction proteins such as claudin-5, occludin, and zonula occludens-1 [75]. These changes are paralleled by the disruption of adherens junctions, including VE-cadherin, which results in barrier impairment and increases paracellular migration. Moreover, parasite-induced signaling via endothelial receptors, such as protease-activated receptors (PARs), leads to cytoskeletal remodeling and dissolution of junctions [76]. Electron microscopic studies also show that infected tissues have swollen endothelial cells, an abnormal shape of tight junctions, and an increase in transcytotic activity [77]. Taken together, these results suggest a model in which trypanosomal cysteine proteases (specifically bruceipain) act on specific endothelial receptors and junctional proteins, leading to BBB dysfunction and subsequent invasion of the CNS.

There is a growing body of experimental and clinical evidence that trypanosomes enter the CNS via the blood-choroid plexus membrane and not directly by penetration of the parenchymal BBB [78]. Earlier inflammation of the choroid plexus is detected by histopathological examination of the choroid plexus through epithelial cell activation, infiltration of leukocytes, and an increase in permeability. This choroid plexitis occurs together with the introduction of the parasites and inflammatory mediators in the CSF, usually before the onset of overt neurological symptoms. The fact that parasites appear early in CSF proves the idea that the ventricular system is an initial staging ground of dissemination of the CNS [79]. In addition to the choroid plexus, trypanosomes demonstrate remarkable neurotropism toward circumventricular organs (CVOs), specialized brain structures lacking a conventional blood-brain barrier. These structures, including the median eminence, organum vasculosum of the lamina terminalis, area postrema, and subfornical organ, possess fenestrated capillaries that may facilitate early parasite entry [80]. Experimental studies have demonstrated that trypanosomes target CVOs early in infection, even before breaching the parenchymal BBB [81], and that these structures become sites of local immune activation with expression of pro-inflammatory cytokines, including TNF-α, IL-1β, and IL-1β converting enzyme [82]. The strategic localization of CVOs adjacent to hypothalamic sleep–wake regulatory centers and circadian networks suggests that early infection and inflammation at these sites could contribute to the initiation of sleep–wake disturbances before widespread parenchymal invasion. This mechanism may explain why typical alterations of sleep architecture can represent early signs of HAT and precede parasite traversal of the BBB [83]. This provides a potential mechanism for the early onset of sleep architecture changes observed clinically.

The so-called Trojan horse hypothesis is one of the suggested mechanisms that help to explain such early invasion: trypanosomes use the help of the host immune cells to obtain a path to the closed compartments [84]. While the precise extent of intracellular parasite transport remains under investigation, the role of immune cell trafficking in facilitating CNS entry is well documented. Monocytes and other leukocytes activated due to systemic infection are also recruited to the choroid plexus and meningeal vasculature. The parasites can stick to these cells or temporarily relate to them, in effect circumventing the barriers of epithelial cells in immune cell traffic. Even though the direct intracellular transmission of viable parasites is still controversial, the mediation by immune cells is likely to augment local inflammation and destabilization of the barriers, which subsequently indirectly enhances the entry of parasites into the CSF [30].

Simultaneously, there is evidence of direct barriers to cell translocation by both transcellular and paracellular pathways by parasites. Trypanosomes are very motile and mechanically active, which allows them to apply mechanical forces on the epithelial and endothelial layers [85]. The transcytosis, proven as uptake of parasites and vesicular transport across barrier cells, has been shown in experimental models, and paracellular migration can be noted after cytokine-induced weakening of tight junctions. The local generation of inflammatory mediators such as TNF-α and IFN-γ can further enhance epithelial permeability, forming a permissive environment for the passage of parasites [62]. After initial entry into the cerebrospinal fluid, neuroinvasion is maintained with progressive disturbance of the parenchymal BBB. Parasites at this stage do not occupy the ventricular or meningeal space anymore, but they penetrate the parenchyma of the brain. This mechanism is closely interconnected with the factors of parasites and the inflammatory reactions of the host. Some of the molecules of the parasite that are involved include cysteine proteases, including bruceipain and *Trypanosoma brucei* cathepsin B (TbCatB), which degrade the extracellular matrix and weaken the structural elements of the neurovascular unit [86]. These proteins have direct effects by weakening the integrity of barriers by attacking basal lamina proteins and junctional complexes.

Simultaneously, trypanosome infection prompts the expression and activation of host matrix metalloproteinases, especially MMP-2 and MMP-9, in the endothelial cells, astrocytes, and leukocytes [87]. These enzymes also cause the degradation of tight junction proteins and extracellular matrix scaffolding, increasing the barrier leakage. Pro-inflammatory cytokine environments, such as interleukins, chemokines, and reactive oxygen species (ROS), activate the upregulation of metalloproteinases and form a positive feedback loop of inflammation and breakdown of the barrier [88]. The key element of this process involves the response of the astrocytes to the inflammatory cues by means of morphological and functional activation. The reactive astrocytes have an altered end-foot coverage of cerebral vessels, hypo- or hyper-regulated release of vasoactive and inflammatory mediators, and decreased endothelial tight junction support [53]. This malfunction of the astrocytes, in combination with the activation of the endothelium and detachment of pericytes, disturbs the coordinated regulation of the BBB. Consequently, the immune cells and parasites have progressively more access to the brain parenchyma, which intensifies neuroinflammation and neuronal stress [89]. This coordinated breakdown of the neurovascular unit represents a multi-phase process whereby parasite-derived proteases, host inflammatory mediators, and cellular dysfunction synergize to compromise barrier integrity [13].

Notably, the HAT barrier disruption is dynamic and progressive with time and location in the brain. Initial alterations are mild and could be reversed, but chronic inflammation causes extensive and long-term barrier dysfunction [90]. Such time flow corresponds to the clinical staging criteria of the disease using cerebral fluid abnormalities and neurological symptoms, which makes barrier pathology central in the course of disease development. Gradual breakdown of CNS barriers not only allows access of parasites but also exposes the brain to persistent peripheral-immune signaling [66]. Barrier integrity begins to decline, and inflammatory mediators and immune cells are more and more able to influence the neural environment, which changes the disease to one of immune-mediated pathology, not of invasion [91]. This shift is the beginning of a period of dominance of neuroinflammation, which contributes to much of the neuropathology in advanced cases of HAT.

## 4. Neuroinflammation and Brain Pathology

**Figure 3:**
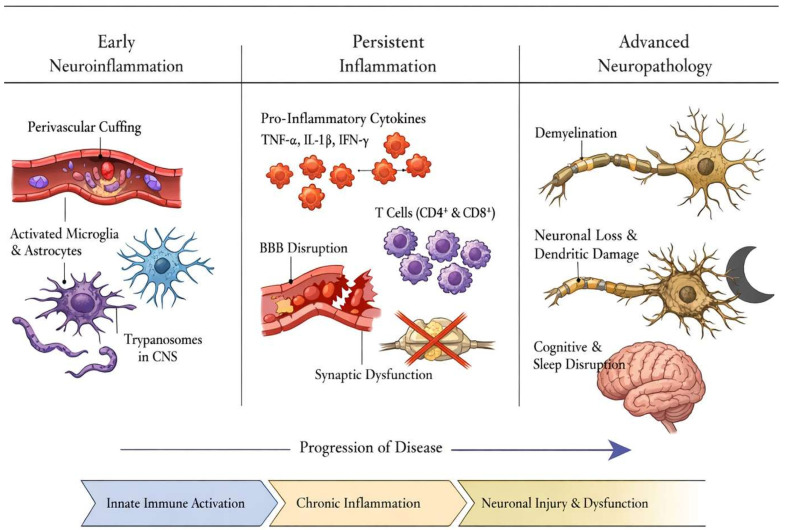
Neuroinflammation and brain pathology in HAT. Figure prepared by the authors with the assistance of generative AI-based drawing tools. All scientific content was conceived, reviewed, and verified by the authors, who take full responsibility for its accuracy.

When the trypanosomes attain persistent access to the CNS, the disease then enters a stage where the parasite persistence is not the only feature of the disease, but a progressive inflammatory response of the host. This is one of the key pathological processes of neuronal dysfunction, white matter damage, and progressive neurological and neuropsychiatric phenotypes in late stages of HAT [42]. Selective vulnerability of the hypothalamus and the SCN in HAT could be attributed to their key function in neuroendocrine and circadian regulation and their increased sensitivity to inflammatory mediators. Inflammatory cytokines like TNF-α, IL-1β, and IFN-γ can cause changes in neuronal signaling in these areas, which can affect molecular clock gene expression and circadian synchronization [92]. Simultaneously, chronic activation of microglia and astrocytes creates a neuroinflammatory environment, affecting not just the functionality of the neurons but also the integrity of white matter. Pro-inflammatory cytokines and ROS can all affect the survival of the oligodendrocytes, reduce the rate of oligodendrocyte progenitor cell (OPC) differentiation, and disrupt myelin maintenance, all of which can lead to widespread dysfunction in neural networks [93]. These are all interrelated inflammatory mechanisms that link parasite-induced neuroinflammation with circadian disruption and progressive neurological pathology. Instead of being an expression of a protective reaction, brain inflammation is dysregulated, chronic, and self-amplifying, and directly leads to tissue injury and functional impairment. The first and the most widespread part of this response is innate immune activation [94]. The immune cells resident in the CNS, microglia, change to an activated state within hours of being exposed to parasite-derived molecules and inflammatory mediators that enter the CSF [95]. Stimulated microglia have hypertrophic morphology, express antigen presentation pathways, and release a wide range of pro-inflammatory cytokines and reactive oxygen and nitrogen species [96]. The exciting reactive transformations also occur in the astrocytes, which usually support neurons and the elements of the BBB by metabolic and structural means. The altered gene expression, failure to control levels of extracellular neurotransmitters, and augmented production of inflammatory intermediates characterize this astrocytosis, which further intensifies the activation of the microglia [97]. Figure 3 depicts the progression of neuroinflammation and resultant brain pathology in HAT, from the early cellular responses to late-stage irreversible neuronal damage.

At the molecular level, the inflammasome signaling pathways are important in the maintenance of the innate immune activation. Pattern recognition receptors found on microglia and astrocytes can sense parasite materials and danger-associated molecular patterns released from damaged host cells and trigger inflammasome complexes and cleavage of pro-inflammatory cytokines to active forms [98]. This action strengthens a local inflammatory environment, which is maintained despite the waning parasite loads, and is an example of the partial uncoupling of the process of inflammation with direct parasite load. With a worsening of barrier integrity and a more pronounced chemokine gradient, adaptive immune cells can have more and more access to the brain. The T lymphocytes, specifically the CD4 + and CD8 + subtypes, invade the mesencephalon and parenchyma, and they play a role in meningoencephalitis [99]. These cells release cytokines that augment the microglial activation and endothelial permeability and have direct cytotoxic effects on infected or stressed neural cells. The B cells and plasma cells are also found in the meninges and the perivascular spaces, where they produce intrathecal antibodies that indicate a local immune response to the presence of parasites but provide inadequate clearance of parasites in the immune-privileged CNS [22].

Although the adaptive immune response is necessary to control the parasites in the body on a systemic level, it is therefore maladaptive in the brain. The continued lymphocytic inflammation maintains tissue stress and inflammation, which encourages neuronal dysfunction as opposed to eliminating infection [100]. This persistent immune pattern, in line with clinical findings that neurological events can still develop despite removal of parasitemia, underscores the importance of host-mediated pathology. The main part of the neuroinflammatory cascade is the uncontrolled synthesis of cytokines and chemokines [101]. Interferon-γ becomes a central mediator, which results in macrophage and microglial activation and the increase in chemokines, which recruit other immune cells across damaged barriers [102]. The TNF-α is one of the factors that cause dysfunction of synapses and neuronal stress, as well as additional impairment of endothelial and glial homeostasis [103]. CXCL10 and other chemokines set up gradients that keep recruiting T cells to the brain, which makes the process of immune cell infiltration and local cytokine release self-perpetuating [104]. Collectively, these mediators generate a long-term inflammatory process that disrupts neuronal signaling, circadian timing, and sleep–wake control.

The combination of such immune processes is manifested in typical neuropathological alterations. Perivascular cuffing, where layers of infiltrating lymphocytes and macrophages are formed around the cerebral blood vessels, is one of the most prominent ones [105]. This is a characteristic of neuroinflammation that indicates the continuity of immune trafficking and local vascular dysfunction. White matter is also an influential feature, as multiple brain areas have been shown to have demyelination and axonal injury [106]. Impairment of oligodendrocytes and myelin sheaths interferes with signal transmission and leads to motor and cognitive impairment and changed sensory processing [107]. Although neuronal injury is not as evident as inflammatory infiltration, it is a key cornerstone of irreversible disease progression. Chronic inflammatory stress on neurons results in dendritic retraction, synapse loss, and metabolic impairment, resulting in death of the cells in extreme instances [108]. Such alterations are especially harmful in circuits that control arousal, cognition, and circadian timing, which gives a mechanistic connection between neuroinflammation and the descriptive clinical signs of late-stage disease.

Notably, HAT is spatially and temporally varying. Initial inflammatory activities can be limited to perivascular and meningeal areas, but in the long-lasting disease, parenchymal damage occurs throughout the disease [77]. This development is similar to clinical staging with references to CSF abnormalities and neurological manifestations, which support the idea of the central position of immune-mediated pathology in disease development. Although it is the neuroinflammation and neuronal damage of the structural and cellular landscape of late-stage disease, the typical exposure of sleep–wake organization is finally manifested on a molecular level in susceptible neural pathways [109]. A combination of inflammatory processes, immune signaling, and persistence of parasites all lead to signaling through circadian timing, arousal, and maintaining a stable sleep state. To provide the relationship between neuropathology and the required behavioral phenotype of HAT, elucidation of these molecular mechanisms is therefore required.

## 5. Molecular Mechanisms of Sleep Dysregulation

The profound changes in sleep–wake structure that are seen in HAT eventually come down to molecular distortions in circadian timing and sleep–wake-state transition circuits. Although previous sections explain the effects of clinical manifestation, barrier pathology, and neuroinflammation, their unification at the molecular level affects the ability to disrupt the main regulators of sleep biology. The sleep homeostasis in this disease is an effect of the interplay between direct parasite-induced and indirect host-mediated inflammatory signaling, which destabilizes circadian pacemakers, neuromodulatory systems, and synaptic homeostasis [12]. Direct impact on sleep control as a pathophysiological element of the disease is becoming an important factor [110]. Sleep–wake impairment in HAT is probably related to a network of molecules rather than to a single molecule. Inflammatory activation promotes tryptophan metabolism through the kynurenine pathway [111], increases nitric oxide production, and stimulates the release of sleep-promoting cytokines, all of which lead to changes in neuronal excitability in the orexinergic and monoaminergic wakefulness-promoting network [112]. The changes lead to sleep disturbances, daytime sleepiness, and a disrupted circadian rhythm. Another possible pathway of chronic neuroinflammation is from reversible functional changes to structural changes in the neurons, which could be useful to investigate the use of markers of axonal injury and disease severity, like neurofilament light chain [113]. Simultaneously, inflammatory mediators such as TNF-α and IL-1β may also interfere with the molecular circadian clock by altering signaling pathways that control the transcription–translation feedback loops of CLOCK, BMAL1, PER, and CRY proteins, further disrupting circadian synchrony and worsening sleep–wake abnormalities [114]. Existing experimental evidence indicates that the disruption of the circadian rhythm associated with HAT is more a consequence of functional impairment of the SCN in early CNS involvement and does not reflect neuronal loss. Experimental work has shown that there is altered expression of c-Fos, disrupted firing patterns of SCN neurons, and abnormalities in the signaling of vasoactive intestinal peptide (VIP) and arginine vasopressin (AVP), which suggests that there is impaired synchronization of the oscillators and modulation of the amplitude of the oscillators [115]. The expression of these genes is likely regulated by neuroinflammatory mediators, such as cytokines and indoles such as tryptophan and metabolites such as kynurenine, which can regulate clock gene expression and affect the transcription–translation loops between CLOCK, BMAL1, PER, and CRY proteins. There is a possibility of a direct effect of molecules produced by the parasite on the core clock mechanism, but such evidence is scarce. Continued inflammation and activation of glia and documented neuronal loss in hypothalamic and circadian regulatory areas in advanced disease may make these initially reversible functional changes more permanent, with circadian and sleep–wake dysfunction [36]. The primary molecular mechanisms driving sleep dysregulation and their functional consequences are summarized in Table 3. The trypanosomes localize in areas including the periventricular and hypothalamic areas and are located closely to the SCN, which is the prime circadian clock of the mammalian brain. Infected animals show abnormal expression of the core clock genes and disturbed rhythms of neuronal firing in the SCN by experimental models [115]. These alterations cannot be explained only by the generalized inflammation, which implies that parasite-derived molecules or metabolic by-products may act directly on the clock machinery, although direct evidence for this remains scarce. SCN ablation disrupts temporal consistency in downstream hypothalamic and brainstem targets, which leads to the loss of hormonal rhythms, sleep–wake reversal, and disrupted vigilance conditions [116]. Beyond functional alterations, structural damage to the SCN has been documented in experimental *T. b. gambiense* infections. Tesoriero et al. (2018) demonstrated approximately 30% neuronal loss in the SCN of infected Mastomys natalensis, affecting both the dorsomedial and ventrolateral subdivisions of the nucleus [47]. This neuronal loss was accompanied by marked astrocyte activation and was specific to *T. b. gambiense* rather than *T. brucei* infection, suggesting differential susceptibility of the biological clock to human-pathogenic trypanosomes. Importantly, this neuronal loss was not observed in other brain regions such as the hippocampal dentate gyrus, indicating selective vulnerability of SCN neurons rather than widespread neurodegeneration [47]. The partial but permanent damage to the SCN may explain why sleep–wake disturbances can persist or only slowly recover even after successful parasiticidal treatment, underscoring the importance of early therapeutic intervention before irreversible neuronal loss occurs.

Simultaneously, trypanosomes drastically change the host tryptophan metabolism, shifting it towards the kynurenine pathway and away from serotonin production. This restructuring of metabolism leads to the production of bioactive tryptophan metabolites such as kynurenine and downstream neuroactive substances. Some of these metabolites permeate the BBB or are produced locally in the CNS, where they induce neuromodulatory and neurotoxic activities [117]. Disturbed serotonin and kynurenine derivative balance is known to interfere with the processes of initiating sleep, REM to non-REM cycling, and regulation of mood, and offers a molecular interconnection between infection, disturbed sleep, and neuropsychiatric symptoms [108]. In addition to direct parasitic effects, the role of inflammatory-mediated mechanisms is the most dominant in the development of sleep pathology [118]. The presence of pro-inflammatory cytokines such as interleukin-1β and TNF-α, which are high in cerebrospinal fluid during cerebral infection, makes them effective somnogens in physiological conditions [119]. With persistent infection, however, they are overproduced, causing pathological fragmentation of sleep instead of the promotion of restful sleep. Such cytokines can change neuronal excitability, adapt synaptic transmission, destabilize the balance between sleep-promoting and wake-promoting brain networks, and produce unstable switches between vigilance states [13].

**Table 3:** Molecular mechanisms of sleep–wake dysregulation in Human African Trypanosomiasis (HAT).

Mechanistic Category	Primary Target/System	Specific Molecular/Cellular Effect	Functional Consequence for Sleep	Evidence Source/Translational Confidence	Key References
Circadian Clock Disruption	SCN and its outputs.	Parasite proximity to the SCN disrupts core clock gene expression.	This leads to a loss of circadian rhythms and sleep–wake reversals.	Animal models (rat, Mastomys), moderate	[120]
Tryptophan Metabolism Shift	Serotonin synthesis and the kynurenine pathway.	Infection diverts tryptophan from serotonin to neuroactive kynurenine metabolites.	The result is disrupted sleep initiation, REM regulation, and mood.	Human and experimental, moderate–strong	[58]
Pro-inflammatory Cytokine Signaling	Neuronal networks governing sleep–wake transitions.	Pathologically elevated CNS IL-1β and TNF-α alter neuronal excitability.	This causes pathological sleep fragmentation instead of physiological sleep.	Human CSF and experimental, moderate–strong	[114]
Aminergic Neurotransmitter Dysfunction	Serotonergic (raphe) and dopaminergic (midbrain) systems.	Cytokines and metabolic shift deplete serotonin and impair dopamine signaling.	Serotonin loss disrupts sleep continuity; dopamine loss causes daytime sleepiness.	Experimental, moderate	[73]
Nitric Oxide (NO) Mediated Disruption	Arousal circuits and mitochondrial function.	Neuroinflammation induces iNOS, leading to chronically high NO levels.	Excess NO inhibits arousal circuits and worsens sleep fragmentation.	Rodent experimental, moderate	[121]
Synergistic Network Dysregulation	Integrated sleep–wake and neuroimmune circuitry.	Molecular pathways (cytokines, NO, metabolites) interact in a feed-forward loop.	Transient disruptions become a chronic, self-perpetuating cycle.	Integrative/inferential, hypothetical	[83]
Clinical and Pathological Implications	The dynamic balance of functional impairment.	Molecular sleep disturbances are partially reversible after treatment.	This indicates a functional pathophysiology prior to irreversible neuronal loss.	Human clinical, moderate–strong	[122]

Transduction of cytokines also disrupts aminergic neurotransmitter systems, which are fundamental in keeping one awake and controlling REM sleep. Serotonergic neurons of the raphe nuclei and dopaminergic neurons of the midbrain are especially susceptible to inflammatory stress [123]. The diminished serotonin supply, which occurs because of the inhibition of generation by cytokines and because of the shift of tryptophan metabolism, disrupts sleep continuation and circadian entrainment. Dopaminergic dysfunction is another factor that leads to excessive daytime sleepiness, poor motivation, and impaired reward processing and increases the neurobehavioral phenotype of late-stage disease [124]. Nitric oxide is another important molecular mediator between inflammation and sleep disturbance [125]. Neuroinflammation increases inducible nitric oxide synthase in microglia and astrocytes and results in prolonged production of nitric oxide. Although nitric oxide is usually involved in synaptic plasticity and sleep regulation, high concentrations have inhibitory effects on neuronal activity and mitochondria. High levels of nitric oxide interrupt communications in arousal-promoting circuits and distort the process of REM to non-REM, which only strengthens the process of sleep fragmentation and circadian instability [126].

Notably, these molecular mechanisms do not act independently, but they interact synergistically to destabilize sleep–wake activity. The metabolism of neurotransmitters is affected by cytokines, clock gene expression is affected by nitric oxide (NO), and circadian signaling is also closely integrated with immune activity [127]. This dysregulation at the level of the network makes short-term sleep disruptions a chronic and self-perpetuating disease comparable to that of disease progression. The reversibility of the sleep abnormalities partially after successful treatment with antiparasitics is indicative that the molecular imbalances are dynamic and not simply degenerative in nature, especially when the intervention is provided prior to massive loss of neurons [128]. The dynamic and reversible character of these molecular disturbances presents a valuable clinical implication: sleep–wake alterations and neuropsychiatric symptoms do not occur directly due to the irreversible loss of neurons but due to the progression of pathological processes according to the parasite load and host defense [129]. This reliance on the stage of disease and inflammatory response is what makes the clear identification of CNS involvement an imperative step in the management of the patient. It is difficult to know when molecular and cellular alterations have exceeded the critical threshold in the perpetuated neuroinvasion, especially with the scattered distribution of parasites and with variability in the extent of neuroinflammation [130]. In turn, the diagnosis of late-stage HAT cannot be based on the clinical signs only but should also incorporate biological markers of the parasite’s presence, immune responses, and barrier disruption in the CNS [131]. These complications highlight the importance of why the parasite is still found in the neural sanctuary, where new advances in cerebral fluid examination, biomarker identification, and supplementary diagnostic methods are still being pursued.

## 6. Challenges in Diagnosing CNS Infection

**Figure 4:**
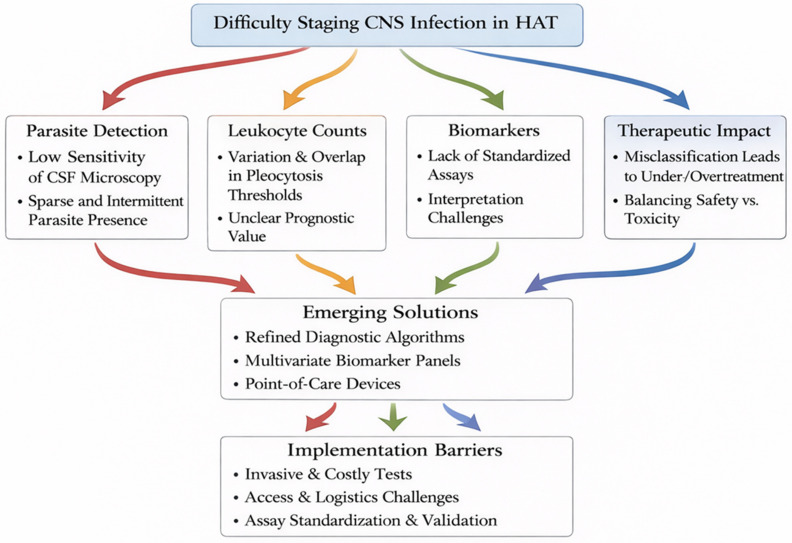
Critical challenges and emerging solutions in diagnosing and staging CNS infection in HAT. Figure prepared by the authors with the assistance of generative AI-based drawing tools. All scientific content was conceived, reviewed, and verified by the authors, who take full responsibility for its accuracy.

One of the most long-standing problems in the management of HAT is the proper diagnosis of the involvement of the CNS. The persistence of neurological and sleep–wake disturbances following successful parasite clearance reflects the complex interplay between reversible functional impairments and irreversible structural damage. While early functional alterations driven by neuroinflammation, neurotransmitter imbalances, and circadian desynchronization may gradually normalize after treatment, the documented neuronal loss in the SCN [47] and potential damage to peptidergic hypothalamic neurons suggest a window of opportunity for intervention. The extent of recovery likely depends on disease stage at treatment initiation, with earlier intervention preserving neuronal populations and maximizing potential for functional restoration. This underscores the critical importance of early diagnosis and staging, as current diagnostic markers may fail to capture the onset of irreversible neural damage. The passage from the hemolymphatic stage to the meningoencephalitic disease is clinically decisive, determining the choice of treatment, prognosis, and toxicity associated with treatment [132]. This transition is hard to delimit since the CNS can serve as a partial refuge whereby the parasites can be sparse, intermittent, and obscured by the host’s inflammatory response. As a result, diagnostic approaches should deal with technical constraints and also with the multifaceted nature of the biology of parasite tolerance and immune stimulation in the CSF and the brain [133]. CSF examination has been considered to be the gold standard of disease staging and confirmation of CNS involvement. CSF trypanosomes can only be clearly seen through direct examination and can be considered an undeniable sign of neuroinvasion, but the sensitivity of this method is intrinsically low [134]. The parasite loads are not large and can be uneven, which leads to frequent false negatives among patients with developed neurological symptoms. Though repeated lumbar puncture and concentration approaches to parasites would be useful in detection, these methods are invasive, resource-intensive, and unworkable in most endemic regions [135]. The primary challenges and emerging solutions in the diagnosis and staging of CNS infection in HAT are outlined in Figure 4.

CSF white blood cell (WBC) counts have been extensively used as an approximation of CNS involvement to counter low levels of detectable parasites. An increase in leukocyte counts indicates that there is meningeal inflammation and is generally associated with the development of the disease [136]. There is much variation in the inflammatory response between different individuals and subspecies of parasites, and there is also excessive overlap between early- and late-stage disease. Neurological symptoms with a moderate amount of pleocytosis in some patients and a high number of WBCs in the absence of distinct CNS pathology are also noted [137]. Depending on fixed leukocyte thresholds thus poses the risk of under- and over-staging, and it has a direct impact on the safety and efficacy of treatment. These deficiencies have led to efforts to identify biomarkers that would better reflect intrathecal immune activation and ongoing neuroinflammation [138]. One of the most promising ones is intrathecal IgM production, which implies the activation of local B cells in the CNS. High levels of CSF IgM are correlated with the stage of disease and parameters of neural involvement and are more sensitive than cell counts. Nonetheless, the elevation of IgM is not specific to the disease and can be affected by co-infections or other inflammatory diseases and therefore does not have great diagnostic value on its own [139].

There has also been an emergence of a strong indicator of CNS involvement in neopterin, a macrophage and microglial activation marker induced by interferon-γ signaling. Neopterin concentrations in the CSF increase at an early stage of neuroinvasion and indicate the severity of the immune response in the brain [140]. Much the same, chemokines like CXCL13, which control B-cell migration and aggregation in inflamed tissues, are also associated with meningoencephalitic disease. High CSF levels of CXCL13 are associated with lymphocytic infiltration and intrathecal antibody, which indicates their usefulness as markers of immune-mediated neuropathology [141]. Though potentially useful, biomarker-based approaches have both practical and conceptual difficulties. The cost, the lack of assay standardization, and the limited availability are some of the impediments to implementation in endemic areas [142]. Further, the majority of biomarkers are evidence of immune reactions of the host as opposed to the presence of the parasite per se, which makes them questionable in terms of specificity to active infection versus low-grade or delayed post-treatment inflammation [143]. The incorporation of multivariate biomarkers into complex diagnostic algorithms can enhance the accuracy; however, these tools need to be validated in different epidemiological contexts.

Leukocyte count in CSF, parasite detection, and neurological examination continue to be the most important tests in the staging of HAT, but sleep and circadian biomarkers may provide additional information about CNS involvement and disease progression. An objective measure, such as actigraphy, could be used to assess sleep fragmentation and circadian disruption in resource-poor settings, and the presence of SOREMPs on polysomnography may be a functional marker of sleep–wake regulatory dysfunction. Likewise, the changes in melatonin patterns, flattening of the cortisol rhythm, and the disruption of core temperature rhythms can be seen as a progressive loss of hypothalamic and circadian regulatory networks [144]. A proposed diagnostic strategy would involve combining traditional parasitological and CSF markers with specific sleep and circadian markers for more accurate, early detection of neuroinvasion, disease staging, and treatment response. As a case in point, patients who have equivocal CSF results, but a high frequency of SOREMPs or circadian disruption, may be followed more closely neurologically and staged again. The incremental diagnostic and prognostic value of these biomarkers is yet to be determined, and prospective longitudinal studies are necessary to verify their sensitivity, specificity, feasibility, and utility in endemic settings prior to their use in routine clinical practice.

**Figure 5:**
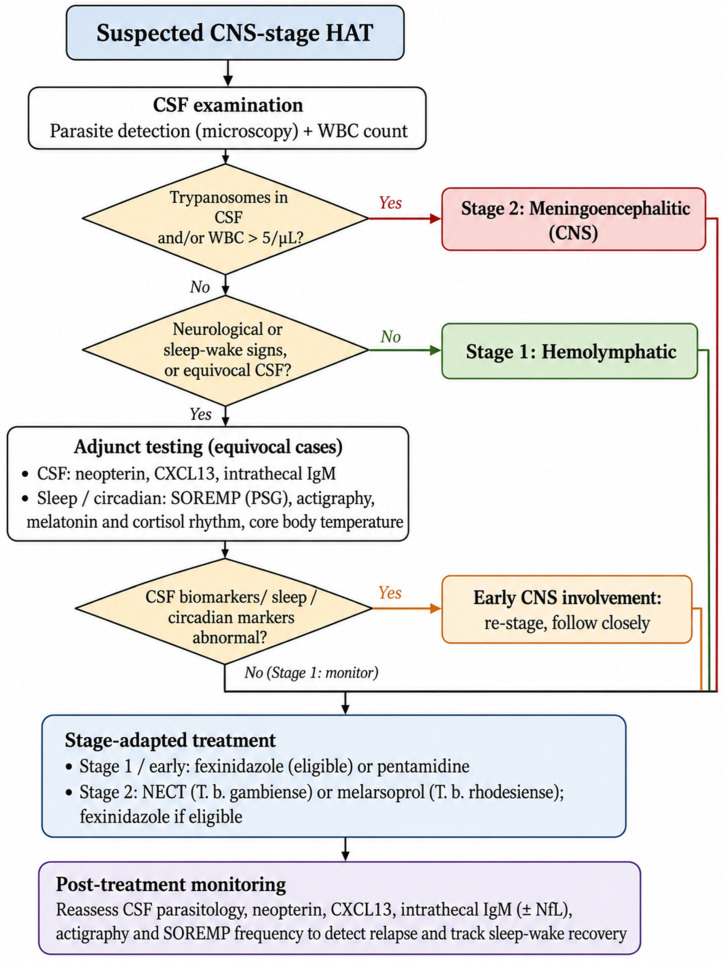
Proposed stepwise decision tree for staging CNS involvement in HAT. CSF microscopy and white blood cell count form the entry point. Cases that remain equivocal are resolved with CSF biomarkers (neopterin, CXCL13, intrathecal IgM) and sleep or circadian markers (SOREMP frequency on polysomnography, actigraphy, melatonin and cortisol rhythms, core body temperature), which guide closer neurological follow-up or repeat staging. Stage assignment then directs stage-adapted treatment, and the same markers are reassessed after treatment to detect relapse and track sleep–wake recovery.

The effectiveness of anti-trypanosomal therapy in reversing TASD is likely influenced not only by parasite clearance but also by the ability of therapeutic agents to reach affected CNS regions and mitigate ongoing neuroinflammation. Figure 5 presents a proposed stepwise decision tree for staging central nervous system involvement in HAT. BBB penetrance is therefore a critical consideration, particularly during the meningoencephalitic stage of infection when sleep and circadian disturbances become most pronounced. While agents such as melarsoprol, eflornithine, and the recently approved oral drug fexinidazole have demonstrated efficacy against CNS-stage disease, their capacity to prevent or reverse established damage to sleep-regulatory circuits remains incompletely understood [145].

Beyond parasite-directed treatment, adjunctive neuroprotective strategies have been proposed, including anti-inflammatory therapies, orexin-based interventions, and melatonin supplementation. However, evidence supporting these approaches remains largely preclinical and should currently be regarded as exploratory. Experimental studies have shown that trypanosome infection disrupts the pineal–SCN axis, alters circadian hormone signaling, and induces structural changes within central circadian networks [44]. Notably, approximately 30% loss of SCN neurons has been reported in animal models, raising important questions regarding the resilience and compensatory capacity of the circadian pacemaker. Although the SCN possesses a degree of functional redundancy, it remains unclear whether the observed neuronal loss exceeds the threshold required to maintain stable circadian output. This uncertainty has direct clinical relevance, as it may help explain the variable recovery of sleep–wake function observed following successful anti-parasitic treatment. Future studies integrating neuroanatomical, electrophysiological, and behavioral assessments are needed to determine whether circadian dysfunction primarily reflects reversible neuroinflammatory processes or irreversible damage to core sleep–wake regulatory circuits.

Table 4 summarizes the diagnostic utility of selected biomarkers, therapeutic characteristics including CNS penetration and relapse rates, and their potential implications for recovery of sleep–wake and circadian disturbances. The table highlights the shortfalls of using conventional CSF parameters, such as finding parasites in CSF and white blood cell counts, as measures of the degree of neuroinvasion. New biomarkers such as CXCL13, neopterin, and intrathecal IgM show potential for good diagnostic performance and may enhance the diagnosis of early involvement of the CNS in conjunction with the standard clinical and laboratory evaluation. These markers call for some caution when the two human-infective subspecies are compared. Most validation work has been carried out in *T. b. gambiense* disease, which runs a chronic course, where CSF neopterin and CXCL13 track the meningoencephalitic stage and the response to treatment [140,146]. Evidence for the acute *T. b. rhodesiense* form is thinner, though not absent. In a study of 85 T. *b. rhodesiense* patients from Malawi and Uganda, intrathecal IgM ranked as the most accurate single staging marker (partial AUC 88%), with MMP-9 (86%) and CXCL13 (85%) close behind, while neopterin fell into a lower-performing group, and three-marker panels reached a partial AUC of 94% [146]. Marker levels may differ across subspecies because disease severity, parasite load, the host inflammatory response, and the timing of clinical presentation are not the same. Performance measured in one form cannot be carried over directly to the other. Multicenter studies that apply matched staging definitions and laboratory methods are needed to confirm biomarker performance in both forms and to show whether subspecies-specific cut-offs are required. The table also shows the current therapeutic options, highlighting differences in CNS penetration, failure rates in therapy, and potential for sleep–wake and circadian disturbances to be influenced by therapy. Together, these data suggest that a multimodal approach to staging, combining clinical data, standard CSF analysis, and biomarker measurements, can help to better classify the disease, select a treatment, and implement earlier intervention before irreversible neurological damage is incurred.

Management of HAT through therapeutic means is closely connected with disease staging, since therapeutic drugs effective against peripheral infection cannot be used in the process of disease management when the parasite has penetrated the CNS [34]. Even minor risks are involved with misclassification: under-treatment leads to further presence of the parasite and neurological damage; overtreatment subjects patients to highly toxic medications [152]. Historically, the primary mode of late-stage treatment was melarsoprol, which had the serious and fatal side effect of treatment-induced encephalopathy [153]. The use of a new nifurtimox-eflornithine combination therapy (NECT) enhanced safety and efficacy but is still logistically challenging [154]. Fexinidazole has more recently revolutionized the therapeutic situation by providing an oral agent that is effective in the treatment of early and selective late disease, but the effects of fexinidazole on late neuroinvasion and long-term neurological outcomes are still under study [145]. Following a successful clearance of the parasite, most patients have long-term neurological sequelae, such as sleep problems, cognitive impairments, and neuropsychiatric disorders. These unsubsidized deficits portray the difference between the cure of parasitology and neurological recovery [155]. Increasing awareness of this gap has provoked the desire to consider adjunctive neuroprotective measures, such as the regulation of neuroinflammation and inhibition of CNS barrier breakdown [156]. It should be noted that barrier pathology does not always correlate precisely with WHO staging criteria, as individual differences in inflammatory responses, parasite burden, and neuroimmune processes may influence the timing and extent of barrier dysfunction, cerebrospinal fluid abnormalities, and sleep–wake disturbances, which do not necessarily progress in parallel. The incorporation of such methods with antiparasitic treatment can eventually yield better results, but additional studies are required to transfer these ideas into clinical practice.

## 7. Conclusions and Future Perspectives

Given the available integrated evidence from this review, we hypothesize that the pathogenesis of HAT sleep–wake syndrome can be divided into three stages. Parasites enter the CNS during the first phase by traversing the choroid plexus and causing early disruption of sleep architecture and neuroinflammation that is localized. In the intermediate stage, chronic inflammation signals lead to disruption of the BBB, infiltration of the parenchyma, and subsequent dysfunction of the orexinergic neuron, monoaminergic arousal systems, and SCN. At the advanced stage, the neuroinflammation, glial activation, white matter pathology, and neuronal loss in circadian and hypothalamic networks cause significant and irreversible sleep–wake and circadian dysfunction. This model shows that there are potential therapeutic windows in the initial stages when the neuroinflammatory process can yet be reversed.

There are currently a number of important questions to be answered. Genetic polymorphisms that have been linked to HAT, including polymorphisms of the APOL1 gene, and polymorphisms of pro-inflammatory cytokine genes, such as the gene for TNF-α, which are associated with differences in disease severity, parasite control, and inflammatory responses may influence the susceptibility of the host to CNS invasion and neurological outcomes of the disease. There is a need for further research to better understand the interaction between these genetic factors, parasite strain diversity, and environmental factors to influence disease progression.

Future treatment should go beyond just eradicating the parasite to specifically target important molecular pathways associated with neuroinflammation and barrier dysfunction. Selective inhibitors of matrix metalloproteinases (MMP-2 and MMP-9) to maintain the integrity of the BBB, modulators of microglial activation to limit chronic neuroinflammation, and orexinergic and circadian neuronal network protection are promising targets. Other strategies, such as melatonin signaling, chemokine pathway modulation, and neuroprotective agents, should be explored as additional therapeutics. A combination of state-of-the-art neuroimaging, single-cell transcriptomics, molecular diagnostics, and longitudinal sleep monitoring will be crucial to differentiate between reversible inflammatory dysfunction and irreversible neurodegeneration and to identify novel therapeutic targets. In conclusion, HAT offers a novel model to explore the intersection of infection, neuroinflammation, and circadian biology and the interplay these elements have on brain function, which could be extrapolated to other infection-induced sleep and neurological conditions.

## Figures and Tables

**Table 1:** Clinical and laboratory features distinguishing *Trypanosoma brucei gambiense* human African trypanosomiasis (TASD/HAT), narcolepsy type 1, and idiopathic hypersomnia.

Feature	TASD (HAT)	Narcolepsy Type 1	Idiopathic Hypersomnia
Excessive daytime sleepiness	Present, progressive	Present, sleep attacks	Present, persistent
Sleep-onset REM periods	Reported	Hallmark (≥2 on MSLT)	Typically absent
Nocturnal sleep	Fragmented, poorly consolidated	Often fragmented	Long, undisturbed, unrefreshing
Circadian rhythm	Disrupted (cortisol, melatonin, temperature)	Usually preserved	Usually preserved
Orexin/hypocretin	Altered via inflammation and neuronal injury	CSF hypocretin is low or absent	Normal
Genetic/HLA	None established	HLA-DQB1*06:02 association	No strong association
CSF findings	Pleocytosis; raised IgM, neopterin, CXCL13	Normal	Normal
Underlying cause	Infection-driven neuroinflammation (secondary)	Autoimmune hypocretin loss	Idiopathic

The asterisk in HLA-DQB1*06:02 signifies a specific allele within the Human Leukocyte Antigen (HLA) system, a standardized nomenclature established by the World Health Organization (WHO) HLA Nomenclature Committee.

**Table 2:** Mechanisms of neuroinvasion in Human African Trypanosomiasis (HAT).

Stage/Mechanism	Proposed Role in Neuroinvasion	Key Findings	Primary Evidence Source	Evidence Level * [Key Reference]
Systemic-to-CNS Transition	Progression from hemolymphatic infection to CNS involvement	CNS invasion appears to occur through a multistep process involving CSF access followed by parenchymal dissemination	Human clinical staging, experimental infection models	Moderate–Strong [65]
Blood–Brain Barrier (BBB)	Restricts the entry of parasites and immune cells into the brain parenchyma	BBB consists of endothelial tight junctions, pericytes, and astrocytic end-feet; relatively resistant during early infection	Neuroanatomical and neuropathological studies	Strong [66]
Blood–CSF Barrier (BCSFB)	Potential initial gateway for CNS entry	The choroid plexus possesses fenestrated capillaries and greater immune surveillance than the BBB.	Histopathology, animal models	Strong [68]
Early Choroid Plexus Invasion	Initial parasite access to CSF compartments	Parasites and inflammatory infiltrates were detected in the choroid plexus and CSF before overt neurological manifestations.	Animal infection models, CSF studies, and post-mortem observations	Moderate–Strong [68]
Trojan Horse Mechanism	Parasite transport via infected leukocytes	Monocytes/macrophages may facilitate parasite trafficking across barriers	Experimental animal studies	Moderate [67]
Direct BCSFB Translocation	Parasite migration through epithelial barriers	Possible transcellular or paracellular passage across the choroid plexus epithelium	Experimental studies	Moderate [62]
Cytokine-Mediated Barrier Alteration	Increased barrier permeability facilitates CNS entry	TNF-α, IFN-γ, and other inflammatory mediators alter barrier integrity and leukocyte recruitment.	Animal models, CSF biomarker studies	Moderate–Strong [69]
Parenchymal Brain Invasion	Spread from CSF to neural tissue	Parasites eventually penetrate deeper brain structures during advanced disease.	Animal models, neuropathology	Moderate [68]
MMP-Mediated BBB Destabilization	Disruption of neurovascular integrity	MMP-2 and MMP-9 contribute to extracellular matrix degradation and junctional dysfunction.	Experimental infection models, biomarker studies	Moderate [70]
Parasite Protease Activity (Bruceipain/TbCatB)	Direct contribution to barrier disruption	Parasite-derived cysteine proteases may facilitate tissue invasion and inflammation.	Experimental studies	Moderate [60]
Astrocyte and Pericyte Dysfunction	Amplification of neurovascular injury	Neuroinflammation may impair astrocyte and pericyte function, further weakening barrier homeostasis.	Experimental models, neuropathology	Moderate [71]
Chronic Barrier Dysfunction	Sustained neuroinflammation and disease progression	Persistent barrier leakage exposes the CNS to peripheral immune mediators and promotes ongoing inflammation	Human pathology, animal models	Moderate–Strong [72]
Immune-Mediated Neurodegeneration	Neurological and sleep-related manifestations	Chronic inflammatory signaling contributes to neuronal dysfunction and disease symptoms.	Human clinical observations, neuropathology, and experimental studies	Moderate–Strong [73]

* Evidence Level Definitions: Strong = supported by multiple complementary human and experimental studies; Moderate–Strong = supported by substantial experimental evidence with some human corroboration; Moderate = primarily supported by experimental models or limited human data; Hypothetical = proposed mechanism with indirect or preliminary evidence.

**Table 4:** Integrated framework for the diagnosis and staging of CNS involvement in HAT, incorporating conventional CSF findings, emerging biomarkers, and treatment options.

Diagnostic Stage	Conventional CSF Findings	Emerging Biomarkers	Diagnostic Performance	Recommended Treatment	Treatment Failure/Relapse Rate	Key References
Stage 1 (Hemolymphatic)	No trypanosomes in CSF; normal or minimally elevated WBC count	Usually low CXCL13, neopterin, and intrathecal IgM	Not routinely required	Fexinidazole (eligible patients)	~2–6%	[147]
Early CNS Involvement	Mild CSF pleocytosis; occasional parasite detection	CXCL13 (among the most accurate single staging markers; partial AUC ≈ 85% in *T. b. rhodesiense*, AUC > 90% in *T. b. gambiense*), neopterin (≈84% specificity at 100% sensitivity; ≈88% correct staging on validation, cut-off 14.3 nmol/L; 79–95% specificity), increased intrathecal IgM (≈86% specificity, ≈92% sensitivity, cut-off 3.4 µg/mL)	Biomarkers improve the detection of early neuroinvasion	Fexinidazole or NECT, depending on disease severity	2–5%	[136,148]
Advanced CNS Disease (Meningoencephalitic Stage)	Trypanosomes in CSF and/or marked pleocytosis	Markedly elevated CXCL13, neopterin, and intrathecal IgM; possible increase in neurofilament light chain indicating neuronal injury	Biomarker panel supports disease staging and prognosis	NECT (*T. b. gambiense*) or Melarsoprol (*T. b. rhodesiense*)	NECT: ~1–5%; Melarsoprol: ~5–10%	[149,150]
Future/Integrated Resource-Limited Algorithm	Standard CSF examination	Sequential use of CXCL13, neopterin, and intrathecal IgM when available	May improve staging accuracy compared with WBC counts alone	Stage-adapted therapy; future role for Acoziborole	Preliminary studies suggest <5% relapse	[148,151]

## Data Availability

No new data were created or analyzed in this study. Data sharing does not apply to this article.
